# Supplementary material for: Effects on maternal and pregnancy outcomes of first-trimester malaria infection among nulliparous women from Kenya, Zambia, and the Democratic Republic of the Congo
Source: PLoS One. 2024 Dec 20;19(12):e0310339. doi: 10.1371/journal.pone.0310339 (PMC11661578; doi:10.1371/journal.pone.0310339)
Supplement: S1 Checklist — (DOCX) [file pone.0310339.s001.docx]

Inclusivity in global research

PLOS’ policy on inclusivity in global research aims to improve transparency in the reporting of research performed outside of researchers’ own country or community and ensures that PLOS publications reporting global research adhere to high standards for research ethics and authorship. Authors of relevant research articles may be asked to complete the questionnaire below, which outlines ethical, cultural, and scientific considerations specific to inclusivity in global research. This questionnaire may be requested when researchers have travelled to a different country to conduct research, if research uses samples collected in another country, research with Indigenous populations or their lands, or if research is on cultural artefacts. Researchers travelling to another country solely to use laboratory equipment will not normally be required to complete the questionnaire. However, the questionnaire can be requested at the journal’s discretion for any submission – if you have been requested to complete this questionnaire by the PLOS journal you submitted to, please do so.

Please complete the questionnaire below and include this as a Supporting Information file with your manuscript. Note that if your paper is accepted for publication, this checklist will be published with your article in the supporting information files. Please ensure that you reference the checklist in the main body of your manuscript. We suggest adding a subsection ‘Inclusivity in global research’ to your Methods section and adding the following sentence: “Additional information regarding the ethical, cultural, and scientific considerations specific to inclusivity in global research is included in the Supporting Information (SX Checklist)”

The questions have been designed to be applicable to a wide range of study types, and there are subsections for both human subjects research and non-human subjects research. If any of the questions are not relevant to your research please mark them as “N/A” as appropriate.

**Ethical considerations, permits and authorship**

*This section is applicable to all research types.*

Provide details as to who granted permissions and/or consent for the study to take place in the Methods section of your manuscript. This should include the names of **all** ethics boards, governmental organizations, community leaders or other bodies that provided approval for the study. If individuals provided approval refer to these people by their role or title but do not list their name(s).

Reported on page number: Page 11 and 12 under ‘Ethics approval and consent to participate’

If there were any deviations from the study protocol after approval was obtained please provide details of these changes in the Methods section of your manuscript.
Did this study involve local collaborators that are residents of the country where the research was conducted or members of the community studied? If you do not have any authors from said communities, please provide an explanation for this below.
Everyone listed as an author should meet PLOS’ criteria for authorship and all individuals who meet these criteria should be included in the author byline, rather than the acknowledgements. For further information please see the journal’s Authorship Policy.

Yes. This sub-study was within a study run by The Global Network for Women’s and Children’s Health Research (Global Network). It is a partnership that includes 8 multidiscliplinary research sites and a Data Coordinating Center. The partnership means that the International PI and the US-based PI are equal partners, and different members of the team take the lead on certain aspects of the studies. This sub-study was conducted in the Democratic Republic of the Congo (DRC), Kenya, Zambia, and Pakistan (data not included from Pakistan) and local collaborators include co-authors from DRC: Antoinette Tshefu, Adrien Lokangaka; from Zambia: Elwyn Chomba, Musaku Mwenechanya; from Kenya: Fabian Esamai and Paul Nyongessa; and from Pakistan: Saleem Jessani and Sarah Saleem.

Reported on page number: N/A

**Human subjects research (e.g. health research, medical research, cross-cultural psychology)**

Did you obtain written informed consent from a representative of the local community or region before the research took place? How did you establish who speaks for the community? Details of written informed consent obtained from study participants should be reported separately in the Methods section of your manuscript.

The partcipants in the trial of low-dose aspirin for the prevention of preterm delivery in nulliparous women with a singleton pregnancy (ASPIRIN trial) ASPIRIN study were recruited using the *Eunice Kennedy Shriver* NICHD Global Network Maternal and Newborn Health Registry (MNHR). Further details of the registry are reported in McClure et al, Reprod Health 2020. The MNHR has been ongoing since 2008 and has ongoing approval from the ethics boards or IRBs within each partner country. At the initiation of the MNHR, approval was sought from the appropriate leader of the participating community. Each site in the ASPIRIN trial established their own plan to screen pregnant women and based their plan on what they thought would be most effective for their community [Hoffman et al., Lancet 2020].

How did members of the local community provide input on the aims of the research investigation, its methodology, and its anticipated outcome(s)?

The international PIs and Country Coordinators are involved in every stage of the research investigation, including the development of aims, methodology and outcomes. The research staff that are involved in data collection and oversight of study procedures are members of the local communities in which they work.

When engaging with the local community, how did you ensure that the informed consent documents and other materials could be understood by local stakeholders?

All consent forms are translated into local language by certified translators and then back translated for accuracy. Informed consent was obtained in the participant’s language of choice, and if the participant was illiterate/unable to sign, their signature was captured by thumbprint.

Will the findings of the research be made available in an understandable format to stakeholders in the community where the study was conducted (e.g. via a presentation, summary report, copies of publications, etc.)? Please provide details of how this will be achieved.

Dissemination meetings will be held in all study sites to explain the results of the findings. Given the ongoing partnership in the NICHD Global Network, these meetings are frequently scheduled with Ministries of Health and local stakeholders to review findings of all research. They take various forms from academic meetings to one-on-one meetings with officials from the Ministries of Health to small group meetings with frontline health care providers.

**Non-human subjects research using specimens/ animals collected as part of the study, or those housed in archival collections. Examples include archaeology, paleontology, botany and zoology.**

Did the permission you obtained from a local authority to perform the study include an agreement on access to outputs and benefit sharing? This may include procedures to enable fair distribution of the benefits and resources arising from the research performed. Please include any details of Prior Informed Consent and Benefit Sharing Agreements obtained. These may be required by field-specific regulations, for example the Convention on Biological Diversity (CBD) and the associated Nagoya Protocol.

N/A

If the material used in your study was imported, please A) provide the year it was imported and B) indicate whether permits were obtained to import/export the materials used, C) provide details of any permits obtained. If this information is not available, please indicate this.

N/A

If you used archival specimens, please state how the material used in your study was acquired by the institute it is held in and provide details of any permits obtained for the original excavations/ sample collection. If this information is not available, please indicate this.

N/A

How was the potential cultural significance of the materials collected in your study to local communities considered in your research design? Were Indigenous peoples and/or local researchers and institutions involved with archaeological excavations / collection of specimens? If so, please provide a description of their involvement.

N/A

If your manuscript includes photographs of human remains please indicate whether authors obtained permission from descendants or affiliated cultural communities to do so.

N/A
