# Supplementary material for: Effects on maternal and pregnancy outcomes of first-trimester malaria infection among nulliparous women from Kenya, Zambia, and the Democratic Republic of the Congo
Source: PLoS One. 2024 Dec 20;19(12):e0310339. doi: 10.1371/journal.pone.0310339 (PMC11661578; doi:10.1371/journal.pone.0310339)
Supplement: S1 Table — (DOCX) [file pone.0310339.s002.docx]

**Supplemental Table 1.** Characteristics of the study participant analysis population, stratified by country

| **Variable** | **DRC** | **KENYA** | **ZAMBIA** |
| --- | --- | --- | --- |
| **Randomized, N** | 484 | 668 | 351 |
| **Maternal age (years), N (%)** | | | |
| < 20 | 409 (84.5) | 312 (46.7) | 204 (58.1) |
| 20-29 | 68 (14.1) | 352 (52.7) | 144 (41.0) |
| > 29 | 7 (1.4) | 4 (0.6) | 3 (0.9) |
| Median (P25, P75) | 18.0 (17.0, 18.5) | 20.0 (18.0, 22.0) | 19.0 (18.0. 21.0) |
| **Projected gestation age at enrollment (weeks, days), N (%) ^a^** | | | |
| 6, 0 - 7, 6 | 50 (10.3) | 115 (17.2) | 40 (11.4) |
| 8, 0 - 9, 6 | 132 (27.3) | 210 (31.4) | 80 (22.8) |
| 10, 0 - 10, 6 | 69 (14.3) | 89 (13.3) | 35 (10.0) |
| 11, 0 - 11, 6 | 82 (16.9) | 88 (13.2) | 50 (14.2) |
| 12, 0 - 13, 6 | 151 (31.2) | 166 (24.9) | 146 (41.6) |
| Median (P25, P75) | 10.7 (9.0, 12.3) | 10.0 (8.3, 11.9) | 11.4 (9.1, 12.7) |
| **Maternal education, N (%)** | | | |
| No formal | 78 (16.1) | 1 (0.2) | 12 (3.4) |
| Primary | 231 (47.7) | 42 (6.3) | 32 (9.1) |
| Secondary | 174 (36.0) | 545 (81.6) | 303 (86.3) |
| University + | 1 (0.2) | 80 (12.0) | 4 (1.1) |
| **Maternal height (cm), mean (SD)** | |  |  |
|  | 155.8 (6.6) | 156.1 (8.9) | 157.5 (6.4) |
| **Maternal weight (kg), mean (SD)** | |  |  |
|  | 50.6 (6.7) | 56.3 (7.5) | 54.6 (9.0) |
| **Maternal BMI (kg/m^2^), mean (SD)** | |  |  |
|  | 20.8 (2.2) | 23.3 (3.5) | 22.0 (3.3) |
| **Antenatal care visits, mean (SD)** | |  |  |
|  | 3.6 (1.3) | 4.2 (1.4) | 3.9 (1.0) |
| **Delivery attendant, N (%)** | |  |  |
| Physician | 11 (2.3) | 29 (4.3) | 26 (7.4) |
| Nurse/nurse midwife | 423 (87.4) | 560 (83.8) | 304 (86.6) |
| Traditional birth attendant | 42 (8.7) | 49 (7.3) | 5 (1.4) |
| Family/Self/Other | 8 (1.7) | 30 (4.5) | 16 (4.6) |
| **Delivery location, N (%)** | |  |  |
| Hospital | 62 (12.8) | 143 (21.4) | 141 (40.1) |
| Clinic/health center | 362 (74.8) | 416 (62.3) | 190 (54.1) |
| Home/Other | 60 (12.4) | 109 (16.3) | 20 (5.7) |
| **Delivery mode, N (%)** | |  |  |
| Vaginal | 467 (96.5) | 628 (94.0) | 323 (92.0) |
| C-section | 9 (1.9) | 24 (3.6) | 18 (5.1) |
| Miscarriage/MTP | 8 (1.7) | 16 (2.4) | 10 (2.8) |

Abbreviations: DRC, Democratic Republic of the Congo; N, number; P25, 25^th^ percentile; P75, 75^th^ percentile; SD, standard deviation; BMI, body-mass index.

^a^ Projected gestational age at enrollment developed from algorithm described in Hoffman et al., 2020.
