# Supplementary figures and images for: Effects on maternal and pregnancy outcomes of first-trimester malaria infection among nulliparous women from Kenya, Zambia, and the Democratic Republic of the Congo
Source: PLoS One. 2024 Dec 20;19(12):e0310339. doi: 10.1371/journal.pone.0310339 (PMC11661578; doi:10.1371/journal.pone.0310339)

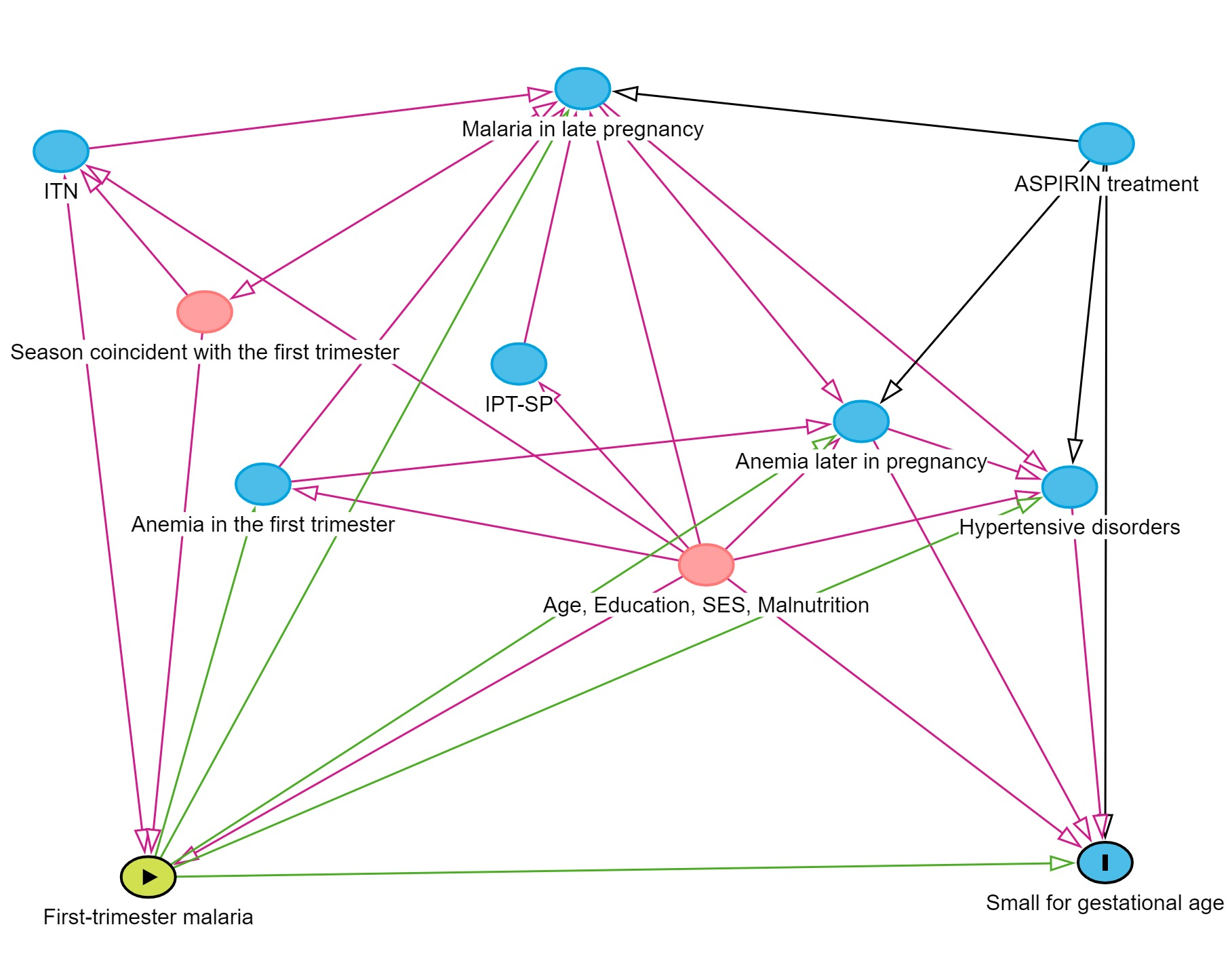

Supplement: S1 Fig — (TIF) [file pone.0310339.s003.tif]

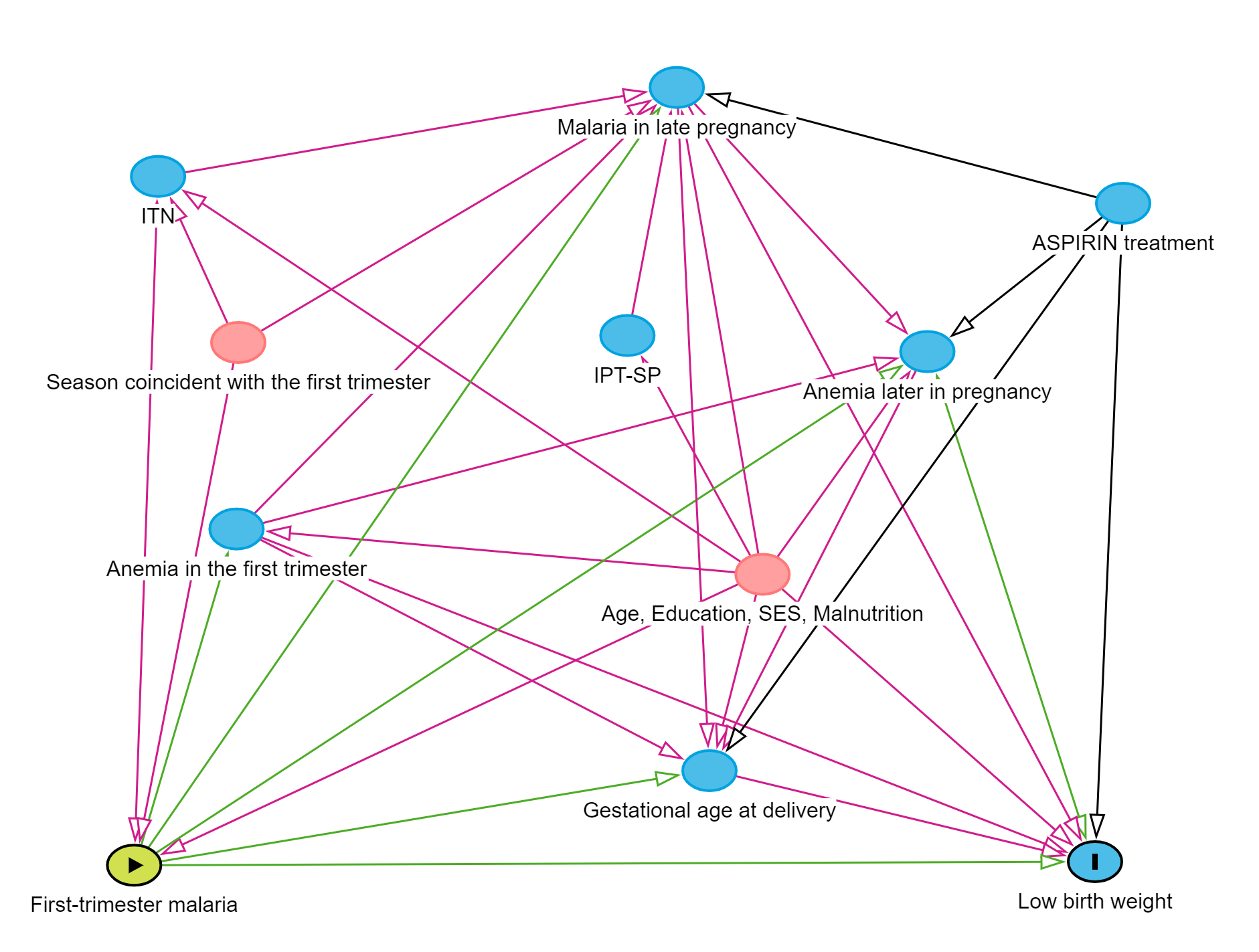

Supplement: S2 Fig — (TIF) [file pone.0310339.s004.tif]

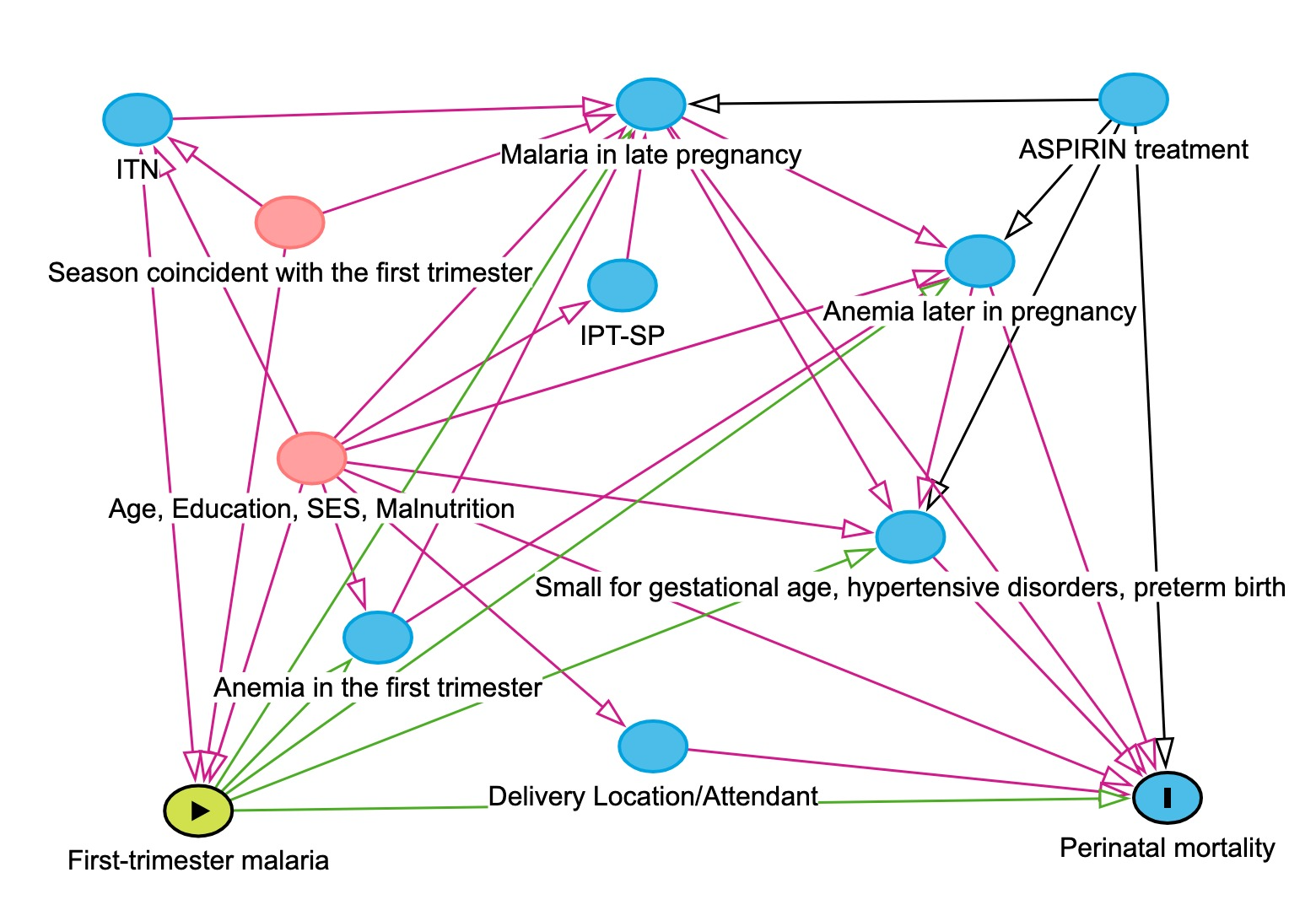

Supplement: S3 Fig — (TIF) [file pone.0310339.s005.tif]

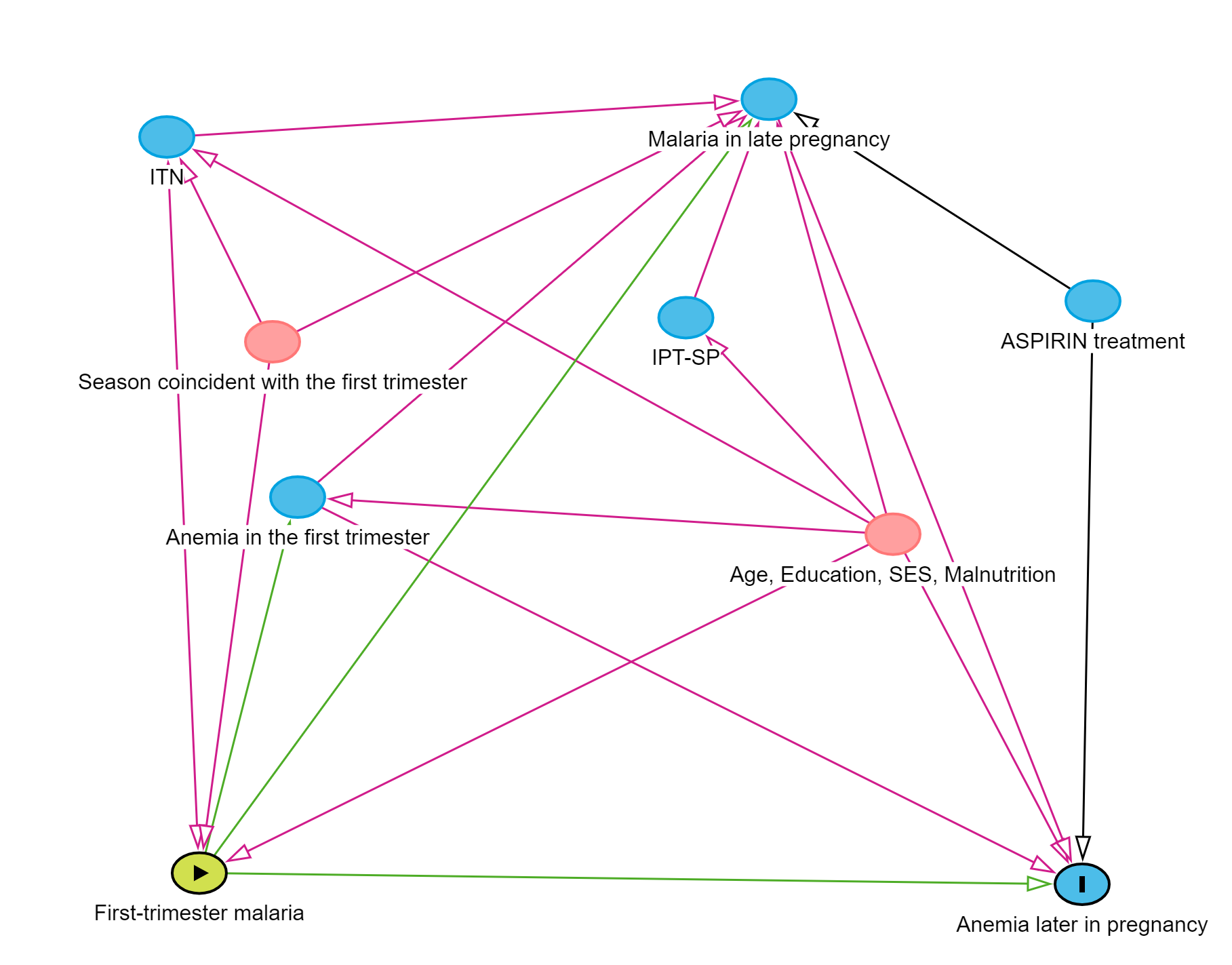

Supplement: S4 Fig — (TIF) [file pone.0310339.s006.tif]
