# Supplementary material for: Effects on maternal and pregnancy outcomes of first-trimester malaria infection among nulliparous women from Kenya, Zambia, and the Democratic Republic of the Congo
Source: PLoS One. 2024 Dec 20;19(12):e0310339. doi: 10.1371/journal.pone.0310339 (PMC11661578; doi:10.1371/journal.pone.0310339)
Supplement: Attachment — Submitted filename: Review_MalariaPregnancy_2may2024.docx [file pone.0310339.s007.docx]

*Effects on maternal and pregnancy outcomes of first-trimester malaria infection among nulliparous women from Kenya, Zambia, and the Democratic Republic of the Congo.*

- Prevalence vs. Incidence in first trimester? Does this make a difference? If you’re including both acute and chronic infection, it may perhaps be masking any effect on pregnancy outcomes
- PCR CT threshold doesn’t seem to have been used, just a “positive” – but using a quantitative test, it might yield more precise results if you look at the difference between CT for incident vs latent infection? Or is this supposed to read “qualitative”, and if so, what is the threshold for determining positive?
  - In your ‘weaknesses’ paragraph of the discussion section, you talk about the underdetection of malaria as a weakness, but it also could have been a strength if you were detecting only incident infections that would be more likely to be associated with poor pregnancy outcomes…. Thinking ahead to policy implications, do you need to ensure you’re most closely estimating malaria incidence in first trimester and how to address that in a context of late ANC initiation in 2^nd^ trimester? Or are you making an argument that all women of reproductive age would benefit from periodic IPTp, and therefore any malaria infection (acute or chronic) is important?
- Tables – please indicate using bold or asterisks the findings which are “significant”
- For Plos One, this is the first paper I’ve reviewed that uses PR and PD – for the sake of readers without much Epi training, a brief explanation in the methods of how to interpret the two may be helpful, including how to interpret CI’s – much as one does for OR’s, where a CI crossing ‘1’ means the findings are not statistically different in exposed than unexposed.
- Anemia in late pregnancy – did your regression analysis control for anemia in early pregnancy? The implication in the narrative is that you’re looking for anemia that developed during pregnancy (an outcome) vs women who were already anemic at enrolment. This seems like a variable you would want to understand a little more that is currently shown
- Your methods aren’t clear about whether the women came from both arms of the parent study, and if so, did you control for aspirin exposure in your analysis? Why or why not?
- Did you assess multicollinearity as well as confounders? Specifically, SES and maternal education?
- Line 231 – what proxy of maternal BMI was used for malnutrition? Be specific. And did you look at overnutrition as well? What was the rationale for not separating nutritional status into three categories? Overnutrition is becoming far more prevalent across Africa, including in rural areas, and has been associated more strongly with poor pregnancy outcomes than undernutrition
- Line 209 – typo ‘wih’ should be ‘with’
