# Supplementary material for: Effects on maternal and pregnancy outcomes of first-trimester malaria infection among nulliparous women from Kenya, Zambia, and the Democratic Republic of the Congo
Source: PLoS One. 2024 Dec 20;19(12):e0310339. doi: 10.1371/journal.pone.0310339 (PMC11661578; doi:10.1371/journal.pone.0310339)
Supplement: Attachment — Submitted filename: Response to Reviewers.docx [file pone.0310339.s008.docx]

Thank you for the opportunity to respond to the academic editor and reviewers. Our responses are in blue.

**Journal requirements:**

When submitting your revision, we need you to address these additional requirements.

1. Please ensure that your manuscript meets PLOS ONE's style requirements, including those for file naming. The PLOS ONE style templates can be found at

<https://journals.plos.org/plosone/s/file?id=wjVg/PLOSOne_formatting_sample_main_body.pdf> and

<https://journals.plos.org/plosone/s/file?id=ba62/PLOSOne_formatting_sample_title_authors_affiliations.pdf>

We would like to thank the reviewer for their request to ensure matching PLOS style requirements and file naming. We have made these changes needed to ensure that we are meeting PLOS ONE style requirements.

2. Please include a complete copy of PLOS’ questionnaire on inclusivity in global research in your revised manuscript. Our policy for research in this area aims to improve transparency in the reporting of research performed outside of researchers’ own country or community. The policy applies to researchers who have travelled to a different country to conduct research, research with Indigenous populations or their lands, and research on cultural artefacts. The questionnaire can also be requested at the journal’s discretion for any other submissions, even if these conditions are not met.  Please find more information on the policy and a link to download a blank copy of the questionnaire here: <https://journals.plos.org/plosone/s/best-practices-in-research-reporting>. Please upload a completed version of your questionnaire as Supporting Information when you resubmit your manuscript.

We would like to thank the reviewer their request to complete a copy on inclusivity in global research in our revised manuscript. We have uploaded a completed version of this questionnaire as our Supporting Information in our resubmission of this manuscript.

3. When completing the data availability statement of the submission form, you indicated that you will make your data available on acceptance. We strongly recommend all authors decide on a data sharing plan before acceptance, as the process can be lengthy and hold up publication timelines. Please note that, though access restrictions are acceptable now, your entire data will need to be made freely accessible if your manuscript is accepted for publication. This policy applies to all data except where public deposition would breach compliance with the protocol approved by your research ethics board. If you are unable to adhere to our open data policy, please kindly revise your statement to explain your reasoning and we will seek the editor's input on an exemption. Please be assured that, once you have provided your new statement, the assessment of your exemption will not hold up the peer review process.

We would like to thank the editor for highlighting the requirement that our data will be available on acceptance. We will comply with the data availability procedures of the NICHD Global Network that include data sharing through the N-DASH system, which is publicly available. The data coordinating center (RTI International, Durham, North Carolina, United States) will prepare the data for upload to N-DASH.

4. Your ethics statement should only appear in the Methods section of your manuscript. If your ethics statement is written in any section besides the Methods, please move it to the Methods section and delete it from any other section. Please ensure that your ethics statement is included in your manuscript, as the ethics statement entered into the online submission form will not be published alongside your manuscript.

We would like to thank the editor for highlighting this change. We have moved the ethics statement to our Methods section (now Lines 263-276) and deleted it from other sections.

5. We note that Figure 1 in your submission contain [map/satellite] images which may be copyrighted. All PLOS content is published under the Creative Commons Attribution License (CC BY 4.0), which means that the manuscript, images, and Supporting Information files will be freely available online, and any third party is permitted to access, download, copy, distribute, and use these materials in any way, even commercially, with proper attribution. For these reasons, we cannot publish previously copyrighted maps or satellite images created using proprietary data, such as Google software (Google Maps, Street View, and Earth). For more information, see our copyright guidelines: <http://journals.plos.org/plosone/s/licenses-and-copyright>.

We require you to either (1) present written permission from the copyright holder to publish these figures specifically under the CC BY 4.0 license, or (2) remove the figures from your submission:

a. You may seek permission from the original copyright holder of Figure 1 to publish the content specifically under the CC BY 4.0 license.

We recommend that you contact the original copyright holder with the Content Permission Form (<http://journals.plos.org/plosone/s/file?id=7c09/content-permission-form.pdf>) and the following text:

“I request permission for the open-access journal PLOS ONE to publish XXX under the Creative Commons Attribution License (CCAL) CC BY 4.0 (<http://creativecommons.org/licenses/by/4.0/>). Please be aware that this license allows unrestricted use and distribution, even commercially, by third parties. Please reply and provide explicit written permission to publish XXX under a CC BY license and complete the attached form.”

Please upload the completed Content Permission Form or other proof of granted permissions as an "Other" file with your submission.

In the figure caption of the copyrighted figure, please include the following text: “Reprinted from [ref] under a CC BY license, with permission from [name of publisher], original copyright [original copyright year].”

b. If you are unable to obtain permission from the original copyright holder to publish these figures under the CC BY 4.0 license or if the copyright holder’s requirements are incompatible with the CC BY 4.0 license, please either i) remove the figure or ii) supply a replacement figure that complies with the CC BY 4.0 license. Please check copyright information on all replacement figures and update the figure caption with source information. If applicable, please specify in the figure caption text when a figure is similar but not identical to the original image and is therefore for illustrative purposes only.

The following resources for replacing copyrighted map figures may be helpful:

USGS National Map Viewer (public domain): <http://viewer.nationalmap.gov/viewer/>

The Gateway to Astronaut Photography of Earth (public domain): <http://eol.jsc.nasa.gov/sseop/clickmap/>

Maps at the CIA (public domain): <https://www.cia.gov/library/publications/the-world-factbook/index.html> and <https://www.cia.gov/library/publications/cia-maps-publications/index.html>

NASA Earth Observatory (public domain): <http://earthobservatory.nasa.gov/>

Landsat: <http://landsat.visibleearth.nasa.gov/>

USGS EROS (Earth Resources Observatory and Science (EROS) Center) (public domain): [http://eros.usgs.gov/#](http://eros.usgs.gov/)

Natural Earth (public domain): <http://www.naturalearthdata.com/>

We would like to thank the editor for their comment on the copyright of Figure 1. We have removed Figure 1 from our manuscript and have updated the following included figure numbers (e.g., Figure 2 is now Figure 1, etc) as needed.

6. Please review your reference list to ensure that it is complete and correct. If you have cited papers that have been retracted, please include the rationale for doing so in the manuscript text, or remove these references and replace them with relevant current references. Any changes to the reference list should be mentioned in the rebuttal letter that accompanies your revised manuscript. If you need to cite a retracted article, indicate the article’s retracted status in the References list and also include a citation and full reference for the retraction notice.

We would like to thank the editor for their comment to review our reference list. We have reviewed our reference list and confirm it is complete and correct.

**Reviewer #1 Comments:**

Reviewer #1: An interesting and well-written paper. Please address the attached comments - these are areas which require additional clarification for the reader unfamiliar with the parent study and perhaps with the statistical methods used.

Effects on maternal and pregnancy outcomes of first-trimester malaria infection among nulliparous women from Kenya, Zambia, and the Democratic Republic of the Congo.

- Prevalence vs. Incidence in first trimester? Does this make a difference? If you’re including both acute and chronic infection, it may perhaps be masking any effect on pregnancy outcomes

We would like to thank the reviewer for their comment on prevalence versus incidence of first trimester malaria. We are using prevalence as we are recording malaria infection at one time point. We have also highlighted that we are specifically using both acute and chronic malaria infection in our exposure variable by adding to the methods section:

Line 151: As we recorded first-trimester malaria infection at one time point, we used prevalence of first-trimester malaria infection as our exposure. We are thus including both acute and chronic malaria infection in our exposure variable, and thus our exposure is the *total* impact of PCR prevalence of first-trimester malaria.

- PCR CT threshold doesn’t seem to have been used, just a “positive” – but using a quantitative test, it might yield more precise results if you look at the difference between CT for incident vs latent infection? Or is this supposed to read “qualitative”, and if so, what is the threshold for determining positive?

We used quantitative PCR techniques as described in Doctor et al., Diagn Microbiol Infect Dis 2016, which references more specifics published in Taylor et al., J Clin Microbiol 2010. Taylor et al found that these quantitative PCR techniques had an average cycle threshold (CT) value of 32.63, and could detect 39 parasites per microliter. Following these results, we qualitatively defined samples as ‘positive’ for *P. falciparum* infection when florescence for both replicates crossed the threshold prior to the 39th cycle, or when one replicate did not amplify and the other crossed the threshold prior to the 39th cycle. We have added more detail (underlined) to our methods section:

Line 148: The main exposure of interest was first-trimester malaria infection, defined as a positive qPCR result (defined as when florescence for both replicates amplified prior to the 39th cycle or when one replicate did not amplify and the other did prior to the 39^th^ cycle) for *P. falciparum* in a sample obtained during the first trimester.

o In your ‘weaknesses’ paragraph of the discussion section, you talk about the underdetection of malaria as a weakness, but it also could have been a strength if you were detecting only incident infections that would be more likely to be associated with poor pregnancy outcomes…. Thinking ahead to policy implications, do you need to ensure you’re most closely estimating malaria incidence in first trimester and how to address that in a context of late ANC initiation in 2nd trimester? Or are you making an argument that all women of reproductive age would benefit from periodic IPTp, and therefore any malaria infection (acute or chronic) is important?

We thank the reviewer for highlighting these important points. We agree that this is a topic that is ripe for future studies and have expanded the conclusions to include these topics. The new text reads:

Line 470: Future studies on malaria in pregnancy should focus on incident and chronic malaria in the first trimester, associations with symptomatic malaria, and initiation and compliance with IPTp programs. An ongoing intervention trial (ClinicalTrials.gov ID: NCT05757167) will assess how screening and treating malaria infection in the first-trimester impacts adverse pregnancy outcomes.

- Tables – please indicate using bold or asterisks the findings which are “significant”

We would like to thank the reviewer for their request to highlight which findings in the tables are ‘significant’. However, as no findings in the tables are significant, we have made no changes.

- For Plos One, this is the first paper I’ve reviewed that uses PR and PD – for the sake of readers without much Epi training, a brief explanation in the methods of how to interpret the two may be helpful, including how to interpret CI’s – much as one does for OR’s, where a CI crossing ‘1’ means the findings are not statistically different in exposed than unexposed.

We would like to thank the reviewer for highlighting the need on how to briefly interpret PR and PD. In addition to highlighting the null value in the caption of Figure 3 (formerly Figure 4), we have also added to our methods section:

Line 204: A null value for prevalence difference (i.e., no difference in women with or without first-trimester malaria) is 0 and a null value for prevalence ratio is 1.

- Anemia in late pregnancy – did your regression analysis control for anemia in early pregnancy? The implication in the narrative is that you’re looking for anemia that developed during pregnancy (an outcome) vs women who were already anemic at enrolment. This seems like a variable you would want to understand a little more that is currently shown

We would like to thank the reviewer for highlighting that our regression analysis did not control for anemia in early pregnancy. We have clarified this in our methods section:

Line 227: In addition, anemia in the first trimester was included in our DAG and was not identified in the minimally sufficient set of confounders, thus we did not adjust for anemia in the first trimester in our analyses.

- Your methods aren’t clear about whether the women came from both arms of the parent study, and if so, did you control for aspirin exposure in your analysis? Why or why not?

We would like to thank the reviewer for their question on clarifying whether women came from both arms of the parent study and if we controlled for aspirin exposure in our analysis. We thus have added:

Line 134: Women were recruited from both arms of the parent study.

Line 226: ASPIRIN trial arm was randomly allocated without respect to malaria in the first trimester status and thus we did not need to control for the effects of the ASPIRIN study protocol.

- Did you assess multicollinearity as well as confounders? Specifically, SES and maternal education?

We would like to thank the reviewer for highlighting our limitation that we did not assess multicollinearity specifically for SES and maternal education. We thus have added to our limitations in the discussion.

Line 458: We also did not assess collinearity including for SES and maternal education.

- Line 231 – what proxy of maternal BMI was used for malnutrition? Be specific. And did you look at overnutrition as well? What was the rationale for not separating nutritional status into three categories? Overnutrition is becoming far more prevalent across Africa, including in rural areas, and has been associated more strongly with poor pregnancy outcomes than undernutrition

We would like to thank the reviewer for their comment on what proxy of maternal BMI was used for malnutrition. This was a typo, and now has been corrected to:

Line 235: ‘malnutrition (used maternal BMI as a proxy)’

We would like to thank the reviewer for pointing out the possibility of overnutrition impacting our results. Within these research sites, the incidence of overnutrition (as measured by BMIs > 25) is very low (other studies report <5% in the DRC [Bauserman et al., Am J Clin Nutr 2021]). Therefore, we are limited in our ability to make conclusions about the impact of overnutrition in any of our models, and restricted these analyses to evaluation of BMI as a continuous variable. We did not look at overnutrition and agree with the reviewer that this is a limitation of our work. We thus have added to our limitations in the discussion:

Line 455: In addition, while we assessed malnutrition (using maternal BMI as a proxy), we did not assess the impact of overnutrition which is associated with adverse pregnancy outcomes (30).

- Line 209 – typo ‘wih’ should be ‘with’

We would like to thank the reviewer for pointing out this typo. We have fixed it in the revised manuscript.

**Reviewer #2 Comments:**

Reviewer #2: Review Leuba et al. First Trimester malaria
This is an important and interesting analysis of the (potential) effects of malaria in the first trimester, as a secondary analysis of trial data. Malaria was measured by PCR in the first trimester, and outcomes examined included prematurity, low birthweight, small for gestational age, perinatal mortality, anemia in late pregnancy, and spontaneous abortion. The manuscript is well written although could occasionally be a bit more concise (e.g. line 306-312).

We would like to thank the reviewer for their comment on paragraph in the manuscript where we could be more concise. We have updated in the revised manuscript recognizing that the DRC site and the Kenyan site had similar outcomes, and thus combined the sentence, with the insertion underlined.

Line 334: In the DRC site and at the Kenyan site, the prevalence of preterm birth, small for gestational age, low birth weight, or of perinatal mortality was similar between women with and without first-trimester malaria infection (**Table 3, Fig 3**).

I only have a few comments.

Maternal haemoglobin concentration was measured between 26-30 weeks gestation, if I understand correctly, but is referred to as anemia in late pregnancy. However, 26-30 weeks is closer to anemia in the 2nd trimester. Although this is more about semantics I am wondering if there is a better way to express this so people do not mix this up with anaemia at the end of pregnancy, e.g. say anaemia later in pregnancy or anaemia in 2nd half of pregnancy?

We would like to thank the reviewer for their comment on expressing maternal anemia more clearly. We have updated the revised manuscript to follow your suggestion of changing ‘anemia in late pregnancy’ to be ‘anaemia later in pregnancy.’

Was setting (urban/peri-urban/rural) considered for inclusion in the daggity diagram? If diagrams were made for the other outcomes as well, perhaps they can be shared in the supplement?

We would like to thank the reviewer for their comment on considering setting in the DAGs. We did not include setting of urban or rural in our DAG. The research units (listed by country) have a mix of rural and urban settings. Women can move between urban and rural settings during their pregnancies as well, a factor that was not measured in this study. We have added this limitation to our discussion.

Line 455: We did not assess setting (rural/urban) in our DAG.

We would like to thank the reviewer for their comment on additional DAGs. We have made DAG diagrams for all the other outcomes, and have now included them in the supplement.

For malaria testing, a sensitive tool was used compared to e.g. a blood smear or rapid diagnostic test. PCR is more likely to pick up asymptomatic malaria, and lower parasite densities. Was there also information on complaints of fever or measured body temperature at enrolment, and have the authors explored if results were the same with a definition of symptomatic malaria? (This type of analysis may only be possible for Kenya and DRC).

We agree with the reviewer of this interesting and important point. Although we attempted to capture data on malaria symptomology, our data collection was imprecise (free text questions on ASPIRIN data forms), and therefore did not yield high quality or consistent data. Therefore, we do not have data on symptomatology included.

The authors used parametric g-computation as statistical tool. It would be nice if the authors can add a line on the advantage of this method compared to a regular (two stage or one stage) meta-analysis.

We would like to thank the reviewer for their request to highlight the strengths of parametric g-computation. Our goal was to estimate the causal effect of first trimester malaria on pregnancy outcomes. In order to interpret these results as causal effects, we would need to assume counterfactual consistency, exchangeability, and positivity [Naimi et al., Int J Epidemiol 2017].

The final assumption of positivity requires a non-zero probability for all values of exposure (i.e., malaria in the first trimester or not) conditional on the variables required for conditional mean exchangeability (which assumes that all confounding has been controlled in the model). In some countries with low malaria prevalence (e.g., Zambia with 6% first trimester malaria prevalence), this could be an issue in semi-parametric methods such as inverse probability weighting [Keil et al, 2021] that are often used in meta-analysis. However, as parametric g-computation can interpolate and extrapolate, we avoid this issue of ‘non-positivity’ analogue of non-sparsity (positivity/non-positivity existing in infinite samples while sparsity/non-sparsity exist in similar finite samples) [Keil et al, 2021].

To highlight that we were able to develop a standardized outcome, and that to interpret our result as the causal effect of malaria in the first trimester on birth outcomes we needed to meet certain assumptions, we have added to the methods:

Line 241: The mean outcome of malaria in the first trimester on the assessed maternal and birth outcome is the weighted average of the mean outcomes for the combination of values for the confounders included (i.e., the standardized outcome). In order to interpret our results as an estimate of the average causal effect, we need to assume counterfactual consistency, exchangeability, and positivity (28).

Note: a dot is missing at the end of a sentence in line 423.

We would like to thank the reviewer for pointing out this typo. We have fixed it in the revised manuscript.
